# Visual and tactile 3D point cloud data from real robots for shape modeling and completion

**DOI:** 10.1016/j.dib.2020.105335

**Published:** 2020-02-26

**Authors:** Yasemin Bekiroglu, Mårten Björkman, Gabriela Zarzar Gandler, Johannes Exner, Carl Henrik Ek, Danica Kragic

**Affiliations:** a University of Birmingham, UK; b KTH Royal Institute of Technology, Sweden; c Peltarion, Sweden; d University of Bristol, UK

**Keywords:** Point cloud, Tactile sensing, Visual depth sensing, Shape modeling, Shape completion, Implicit surface, Gaussian process, Regression

## Abstract

Representing 3D geometry for different tasks, e.g. rendering and reconstruction, is an important goal in different fields, such as computer graphics, computer vision and robotics. Robotic applications often require perception of object shape information extracted from sensory data that can be noisy and incomplete. This is a challenging task and in order to facilitate analysis of new methods and comparison of different approaches for shape modeling (e.g. surface estimation), completion and exploration, we provide real sensory data acquired from exploring various objects of different complexities. The dataset includes visual and tactile readings in the form of 3D point clouds obtained using two different robot setups that are equipped with visual and tactile sensors. During data collection, the robots touch the experiment objects in a predefined manner at various exploration configurations and gather visual and tactile points in the same coordinate frame based on calibration between the robots and the used cameras. The goal of this exhaustive exploration procedure is to sense unseen parts of the objects which are not visible to the cameras, but can be sensed via tactile sensors activated at touched areas. The data was used for shape completion and modeling via Implicit Surface representation and Gaussian-Process-based regression, in the work “Object shape estimation and modeling, based on sparse Gaussian process implicit surfaces, combining visual data and tactile exploration” [3], and also used partially in “Enhancing visual perception of shape through tactile glances” [4], both studying efficient exploration of objects to reduce number of touches.

**Table:** Specifications Table

Subject	Artificial Intelligence
Specific Subject Area	3D Shape Modeling and Completion
Type of Data	Matrices including 3D point coordinates as .mat files and object scans as .obj files
How data were acquired	The data was acquired using two robot setups. The first robot is composed of a 6-degree-of-freedom KUKA arm, a three-finger Schunk Dextrous Hand (7 degrees of freedom) equipped with tactile sensing arrays and a Kinect stereo vision camera. The second robot is a PR2 robot. ROS was used in programming robot motions, communication and data recording. The data was acquired by letting the robot hands touch the experiment objects at predefined locations and recording tactile and visual measurements from tactile sensors on fingers and Kinect cameras, in the form of 3D point clouds, which later on were mapped to the same reference frame based on camera calibrations and registration to initial frame. We also provide 3D scans of objects for comparing shape approximations from real sensory data to ground truth.
Data format	Raw
Parameters for data collection	The explorative touch locations were discretized, given a fixed object pose, i.e. we used a fixed number of approach directions and heights to touch objects.
Description of data collection	The robots touch the experiment objects in a predefined manner at various exploration configurations and gather visual and tactile points in the same coordinate frame, based on calibration between the robots and the used cameras and registration to initial frame.
Data source location	KTH Royal Institute of Technology, Stockholm, Sweden
	Repository name: Visual and Tactile 3D Point Cloud Data from Real Robots for Shape Modeling and Completion
Data accessibility	Data identification number: DOI: https://doi.org/10.17632/ztkctgvgw6.1
	Direct URL to data: https://data.mendeley.com/datasets/ztkctgvgw6
Related Research Article	G. Zarzar Gandler, C. H. Ek, M. Björkman, R. Stolkin, Y. Bekiroglu, Object shape estimation and modeling, based on sparse Gaussian process implicit surfaces, combining visual data and tactile exploration, Robotics and Autonomous Systems, 2020, doi: https://doi.org/10.1016/j.robot.2020.103433.

**Table:** 

**Value of the Data** • Robotic applications often require perception of object shape information [1], [2] extracted from sensory data that can be noisy and incomplete. This is a challenging task and in order to facilitate comparison of different approaches for shape modeling (e.g. surface estimation), completion and exploration we provide real sensory data (3D point clouds from visual and tactile sensors) acquired from exploring various objects of different shape complexities. • In contrast to currently available datasets, which are to a large extent synthetic, the presented dataset is obtained using two different real robot platforms with different end effectors and tactile sensors, providing variety in sensory data in terms of resolution and the type of objects. • The data also includes ground-truth scans for benchmarking different approaches in comparison to full observations. • Unknown objects can be represented via point clouds estimating unobserved areas [5] or fitting continuous surfaces [6], e.g. Implicit Surfaces based on regression via Gaussian Processes [3,4,[7], [8], [9]] or deep neural networks [10]. The data introduced in this paper was used for building Gaussian Process Implicit Surfaces [3,4] for efficient shape estimation. Following the findings of these studies, the dataset can be used for further development of new shape representations, modeling or exploration.

## 1 Data description

The dataset includes visual and tactile readings in the form of 3D point clouds obtained using two different robot setups that are equipped with visual and tactile sensors. It contains three different files for every object, which include a point cloud from the vision sensor, a point cloud from the tactile sensors and the ground-truth object scan. The data files containing point clouds use the .mat format, i.e. they are MATLAB formatted files, while the ground-truth object scans are .obj files, representing the 3D geometry of objects. The data files are available at a Mendeley data repository [11] , which includes these three files for every object:

• <object_name>_v.mat, containing the visual data (*n* x 3 double, where *n* is the number of visual points for this object),
• <object_name>_t.mat, containing the tactile data (*m* x 1 cell, with each element being *k*_*i*_ x 3 double, where *m* is the number of touches for this object and *k*_*i*_, the number of tactile points for touch *i*).
• <object_name>_scan.obj, containing the scanned object,

Where <object_name> can be box1, box2, box3, cyl1, cyl2, cyl3, cyl4, spray1, spray2 or spray3, for the experiments with the first robot, and box1, box2, box3, cyl1, cyl2, cyl3, cyl4, bottle1, bottle2 or bottle3, for the experiments with the second robot. Visual and tactile points are defined in the same frame.

## 2 Experimental design, materials, and methods

The data was acquired by using two different robot setups. The first robot is composed of a 6-degree-of-freedom KUKA arm, a three-finger Schunk Dextrous Hand (7 degrees of freedom) equipped with tactile sensing arrays and a Kinect stereo vision camera. The robot can acquire tactile imprints via pressure sensitive tactile pads mounted on the Schunk hand's fingers. Each finger of the hand has 2 tactile sensor arrays composed of 6 × 13 and 6 × 14 cells, which yields at most 486 tactile points after one touch. For each touch, the hand is set to a fixed initial joint configuration, where the thumb opposes the other two fingers, then fingers are closed until contact is sensed. The ten objects were placed on a table-top with the Kinect camera overlooking objects from one side.

**Fig. 1:**
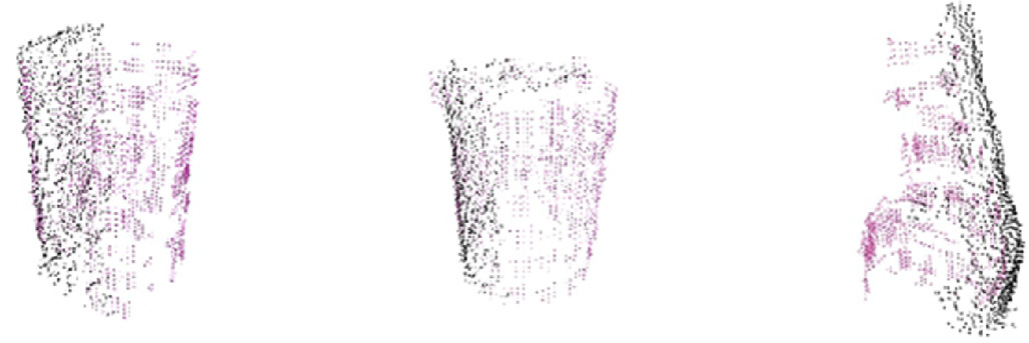
Example readings from objects in the dataset, for the first robot setup. Tactile and visual readings are plotted in red and black, respectively, for box1, cyl1 and spray1. (For interpretation of the references to color in this figure legend, the reader is referred to the Web version of this article.)

An observed object is segmented from its background using a segmentation and tracking system. The system uses stereo vision, the Kinect camera, in a heterogeneous Markov-Random-Field-based framework [12], which uses color and depth information to divide the scene into either planar surfaces, bounded objects or uniform clutter models. From the resulting object segments we get point clouds that serve as starting points for object modeling. To fully cover an object with tactile measurements, up to 54 touches (27 for cyl3 and 18 for box2 due to their lower heights) were performed from the side parallel to the table in a grid of 9 approaching angles (22.5° apart) and 6 heights (spaced at a vertical distance of 2 cm) with respect to the table. The tactile measurements are illustrated as red points in Fig. 2 in Ref. [3]. In order to minimize the potential displacements that can be caused by object movements after touching, before and after a touch, point clouds were registered using the Iterative Closest Point algorithm [13] and measurements were transformed to the initial object frame. Example readings from 3 objects in the dataset can be seen in Fig. 1.

The second robot is a PR2 equipped with two fingers and tactile pads as shown in Fig. 3 in Ref. [3]. The robot hand was guided to touch the objects at different locations to gather tactile observations. The action space was defined by 9 different heights (with a spacing of 2 cm) and 7 different approaching angles (approaching objects from angles between −60° and +60° with a spacing of 20°). Thus at most 63 tactile readings were recorded, complementing the original visual data. For many objects fewer touches were applied due to their size, e.g. box1 with the fewest touches (21 in total). Details about the objects used in the experiments are given in Table 1. Note that objects belong to three different shape categories, namely boxes, cylinders and spray bottles or bottles. We also provide scans of the objects using a Makerbot Digitizer [14].

**Table 1:** Details about objects used in experiments with the first (left) and second (right) robot setups, including i.a. Object names and the number of visual and tactile points.

Name	Object	# of sensory points		# of vertices	Name	Object	# of sensory points		# of vertices
		Visual	Tactile	in scans			Visual	Tactile	in scans
box1		6979	1714	102011	box1		1919	190	35681
box2		5450	445	71097	box2		3113	207	48960
box3		12620	1952	167970	box3		3911	524	79532
cyl1		5029	1580	82930	cyl1		3290	323	64150
cyl2		4528	1460	84755	cyl2		5465	676	95832
cyl3		2765	829	48739	cyl3		3948	620	71019
cyl4		5071	1975	95008	cyl4		4431	737	96935
spray1		4252	1214	94658	bottle1		3759	478	82667
spray2		4084	1508	92439	bottle2		3044	327	83357
spray3		2937	1166	63231	bottle3		2915	321	63770
